# Detecting strawberry diseases and pest infections in the very early stage with an ensemble deep-learning model

**DOI:** 10.3389/fpls.2022.991134

**Published:** 2022-10-12

**Authors:** Sangyeon Lee, Amarpreet Singh Arora, Choa Mun Yun

**Affiliations:** ^1^ Department of Bio and Brain Engineering, Korea Advanced Institute of Science and Technology (KAIST), Daejeon, South Korea; ^2^ Sherpa Space Inc., Daejeon, South Korea

**Keywords:** strawberry disease, pest infection, deep learning, early diagnosis, image analysis, data acquisition system

## Abstract

Detecting early signs of plant diseases and pests is important to preclude their progress and minimize the damages caused by them. Many methods are developed to catch signs of diseases and pests from plant images with deep learning techniques, however, detecting early signs is still challenging because of the lack of datasets to train subtle changes in plants. To solve these challenges, we built an automatic data acquisition system for the accumulation of a large dataset of plant images and trained an ensemble model to detect targeted plant diseases and pests. After obtaining 13,393 plant image data, our ensemble model shows a decent detection performance with an average of AUPRC 0.81. Also, this data acquisition and the detection process can be applied to other plant anomalies with the collection of additional data.

## Introduction

As many artificial intelligence techniques are applied to various fields, the agricultural science field is no exception. For example, computer vision methodologies are introduced to various plant image analysis tasks, like plant classifications (Barre et al., 2017; Wäldchen and Mäder, 2018) and plant diseases and pests detections (Shruthi et al., 2019; Chouhan et al., 2020). Plant diseases and pests detection is one of the most actively studied areas with deep-learning-based computer vision techniques because they overcome the challenging points of traditional computer vision models, in the point that deep-learning-based models do not need any human-guided image features and are less affected by environment variations with a large dataset.

Strawberry (*Fragaria × ananassa*) is one of the most economically important crops produced commercially in 76 countries (Simpson, 2018). China is the world’s largest producer of strawberries, but South Korea and Japan too are considered big producers in East Asia owing to their ideal environmental conditions conducive for producing high quality strawberries (Simpson, 2018). However, this prominent and exotic fruit known for its rich nutritional value and flavors, is prone to damage due to strawberry disease and pests leading to enormous economic damage every year (Sampson et al., 2016; Perasch et al., 2019). Traditionally number of plant diseases were identified and diagnosed by visually analyzing the field/crops by an experienced farmer or by laboratory inspection of the sample. This generally require vast expert knowledge and high costs. Moreover, neither method is particularly effective, with carrying a high probability of failure in successfully diagnosing specific diseases, leading to erroneous conclusions and treatments (Ferentinos, 2018).

Detecting plant diseases in their earliest stages can reduce the amount of potentially harmful remedial chemicals to treat them and lower labor costs for managing damaged plants. As many greenhouses are quite large, it is not always easy for even the most experienced farmers to identify plant diseases before they have spread. For this reason, an automated disease detection process will prove to be a valuable supplement to the labor and skill of farmers. Early detection and correct recognition of pest is very essential to not only prevent crop damage but also prevent incorrect and excessive use of pesticide sprays (Dong et al., 2021). Based on our 6 years of strawberry research and experiences of our partner strawberry research institutes, we identified powdery mildew, spider mite and calcium deficiency as the three most common pest/disease problems encountered in strawberry cultivation.

**Figure 1:**
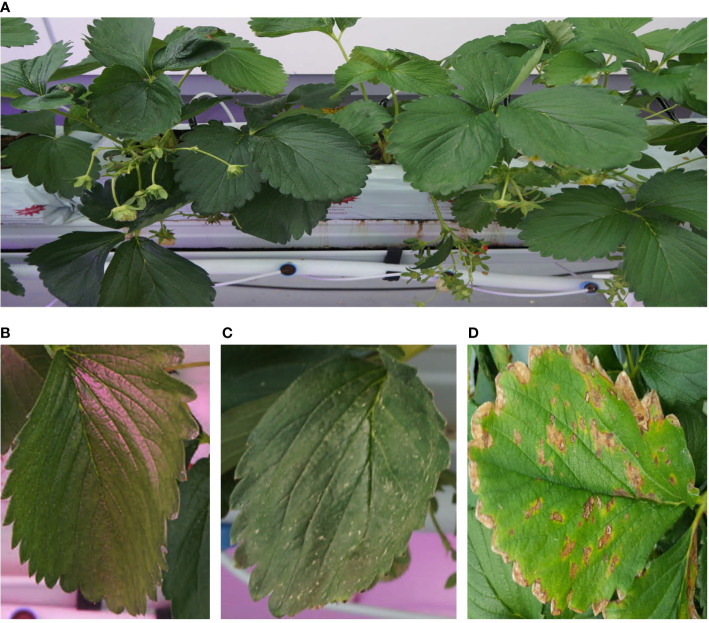
Example of images **(A)** An example of image taken from the image acquisition system, **(B)** a normal leaf image, **(C)** a leaf infected by spider mite. In initial stage, spider mite makes small yellow spots on the surface of leaves. **(D)** An example of image provided by PlantVillage database (David. et al., 2016). Images from the database contain infected leaves in severe phase of diseases or pests.

As aforementioned, quick responses to early signals of plant diseases and pests are very important to minimize their damage from them. However, early detection of those signals is difficult because of the subtlety of the features of symptoms and the difficulty in data collection. While there are many studies on strawberry diseases or pests detection tasks, aiming at various strawberry diseases and pests such as leaf scorch (Ferentinos, 2018), powdery mildew (Kim et al., 2021; Shin et al., 2021; Xiao et al., 2021), verticulum wilt (Nie et al., 2019), gray mold (Nie et al., 2019; Kim et al., 2021; Xiao et al., 2021; Bhujel et al., 2022), and so on, using deep-learning-based computer vision techniques, most of these works are focused on detection itself, not for early detection. Most of these kinds of studies used plant image datasets containing plant images with somewhat progressed diseases acquired from public plant image databases such as PlantVillage (David. et al., 2016) or relatively small size of the dataset under few thousand images which is not suitable to cover environment variations such as lumination, weather, and many interrupting objects. Also, most of these images show considerably progressed symptoms such as severe leaf wilting, brown necrotic lesions, and chlorosis. But these symptoms are not significant in their earliest stages and many plant diseases or pest datasets cannot cover them (**Figure 1**).

After all, the main challenging point of the early detection of plant diseases and pests is the difficulty of data acquisition. Large-scale and high-quality data is a key element of machine learning techniques, but it is difficult to accumulate huge amount of plant image data in various conditions and various life stages manually because diseases and pests progress quickly from the early phase. To overcome these challenges, in this research, we developed a strawberry plant imaging system to automatically acquire large image data of strawberry plants and an artificial intelligence model to detect early symptoms of strawberry diseases or pests using the acquired images. With an automatic image data acquisition system and artificial intelligence-based object detection model, we collected a large amount of strawberry plant image datasets in various conditions and trained a detection model to find symptoms of seven plant diseases and pests in its early stage. Also, to enhance the performances and the robustness of an object detection model, we trained several models and aggregated their outputs in the manner of ensemble methodologies (Korez et al., 2020; Xu et al., 2020). Our deep learning-based ensemble model shows that it can detect those anomalies with decent performance, which an AUPRC value over 0.65 for every case. This system may enable rapid responses to strawberry plant diseases and pests before they progress to more severe stages.

## Materials and methods

### Data acquisition

**Figure 2:**
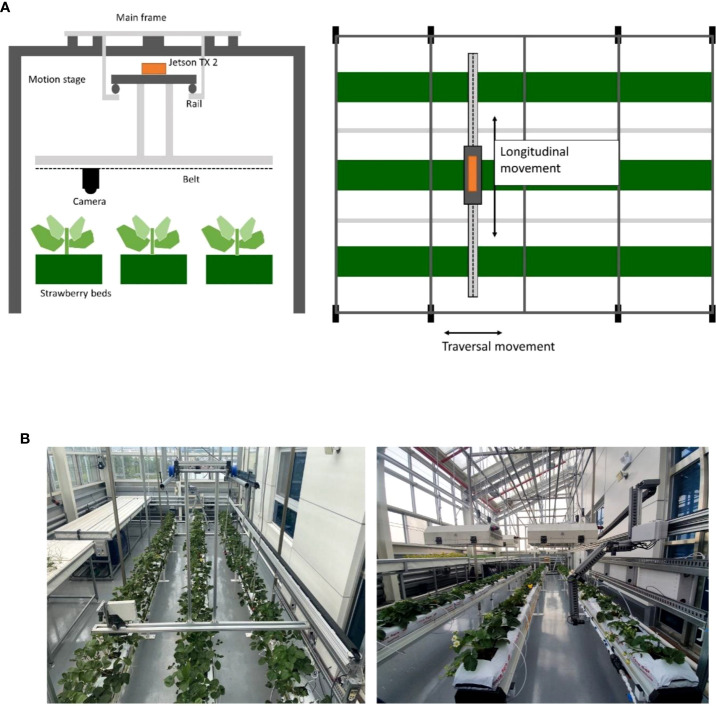
Overall information on the data acquisition system. **(A)** A vertical schematic of the system and a horizontal schematic of the system, **(B)** Actual view of the installed data acquisition system.

As aforementioned, it is essential to collect large plant image data to train an artificial intelligence model to detect early signs or symptoms of plant diseases or pests. However, it is challenging to collect a large amount of plant image data for a long time and under various conditions of plants and environments. To overcome this problem, we built a data acquisition system to automatically produce a large amount of strawberry image data. We built a frame structure to take photos of strawberry plants periodically. (**Figure 2**; **Supplementary Figure 1**) **Figure 2A** shows the cross-sectional schematic diagram of the system. The structure consists of the main frame covering three beds of strawberries, and a rail-based motion stage with a step motor (MS17HDBP4100) that allows its traversal movement. We connected the ELP 8MP USB camera, which can take images with resolution 3265*2448, under the motion stage. The camera is connected to the timing belt for a longitudinal movement. For further work, we attached other sensors for infrared light (VL53L0X V2) and ultrasonic (HC-SR04), but they are not used in this study. The device is controlled by a Jetson TX2 installed on the motion stage. It has a built-in CPU and GPU so it is suitable for NVIDIA AI toolkits, and also shows high FLOPs compared to similar board computers. There are four grid parameters used to control the motor. Parameter *x* and *y* represent the length of the strawberry bed and the length of the width of the system respectively, and *dx* and *dy* represent the size of a grid. The control program sends the command to move the motor, estimates the travel time of the camera, and waits until the travel ends before it activates the camera so that it can take pictures while still. The travel time is estimated as the sum of acceleration time, time in the peak velocity and deceleration time. We assumed the time of acceleration and deceleration are equal. Then, we can calculate the travel time needed to move a certain distance. After the travel ends, it takes images of the plant using a connected camera and transfers them to our data storage server while moving both longitudinal and traversal ways. **Figure 2B** shows the real look of the imaging device.

### Data labeling

**Figure 3:**
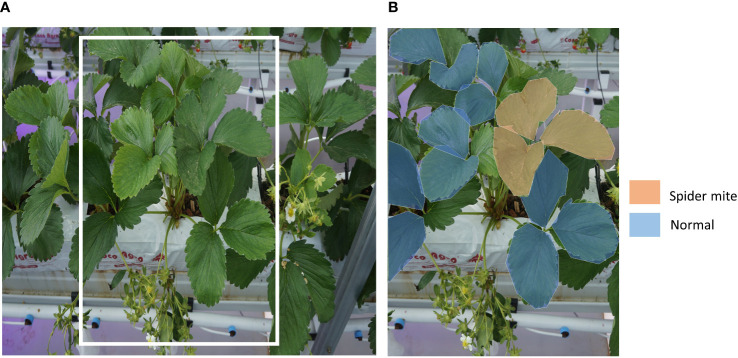
An example of strawberry plant image data. **(A)** A raw image from the data acquisition system. Yellow boxed part is for an example of data labeling. **(B)** An example of labeling of normal leaves and leaves with early symptoms of spider mite.

From the aforementioned data acquisition system, we obtained images of strawberry plants on the raised bed. (**Figure 3A**) To make use of the acquired data, we created a gold standard dataset with plant biology experts. Images were manually annotated through VGG Image Annotator (VIA) (Dutta and Zisserman, 2019) by experts one and one. For every image, regions of observable leaves, flowers, and berries are annotated. The regions of objects on the image were segmented as the polygon shape, then their status was annotated. We segmented all normal and abnormal flowers, berries, and leaves (**Figure 3B**). These polygon-shaped annotations are converted to bounding boxes for the training.

For disease and pest detection, we annotated gray mold, powdery mildew, and blossom blight from berries and angular leafspot, powdery mildew, calcium deficiency, and spider mites from leaves. We determined these abnormal classes following a guideline (**Table 1**)

**Table 1:** Criteria for disease/pest labeling for each disease/pest.

Type	Stress Identification	Criteria of identification/Symptoms	Reference
Disease/Pest	Powdery mildew	Early sign included leaf cupping followed by white powdery mist on the leaves, Appearance of pink petals during flowering and white mist on fruits during fruit stage	(Hall Avice et al., 2017, Aileen, 2015)
	Spider mites	Yellow/Light green color small spots on the leaf surface Webbing around the leaves at later stages	(Aileen and John, 2014)
	Angular leafspot	Angular Shape spots and reddish-brown on the upper leaf surface and water-soaked on the lower leaf surface. Spots look like tiny translucent windows when the leaf is held against the light	(Reddy, 2016, Peres Natalia et al., 2005, Liberato et al., 2006)
	Blossom blight	Grey fungus formed on the stigma leading to blossom blight Black rot and necrosis of entire flower observed at later stage	(Nam et al., 2015, Gubler et al., 1999),
	Grey mold rot	Light brown rot may appear first in area of cap during early stage Fruits get covered by a gray, dusty powder	(Steven, 2020)
Nutrient Deficiency	Calcium deficiency	Appearance of Tip Burn or browning of leaves at the edges Leaf Blade is Crinkled, occurs when leaves grow rapidly/during vegetative phase	(Yara UK limited, 2022)

**Figure 4:**
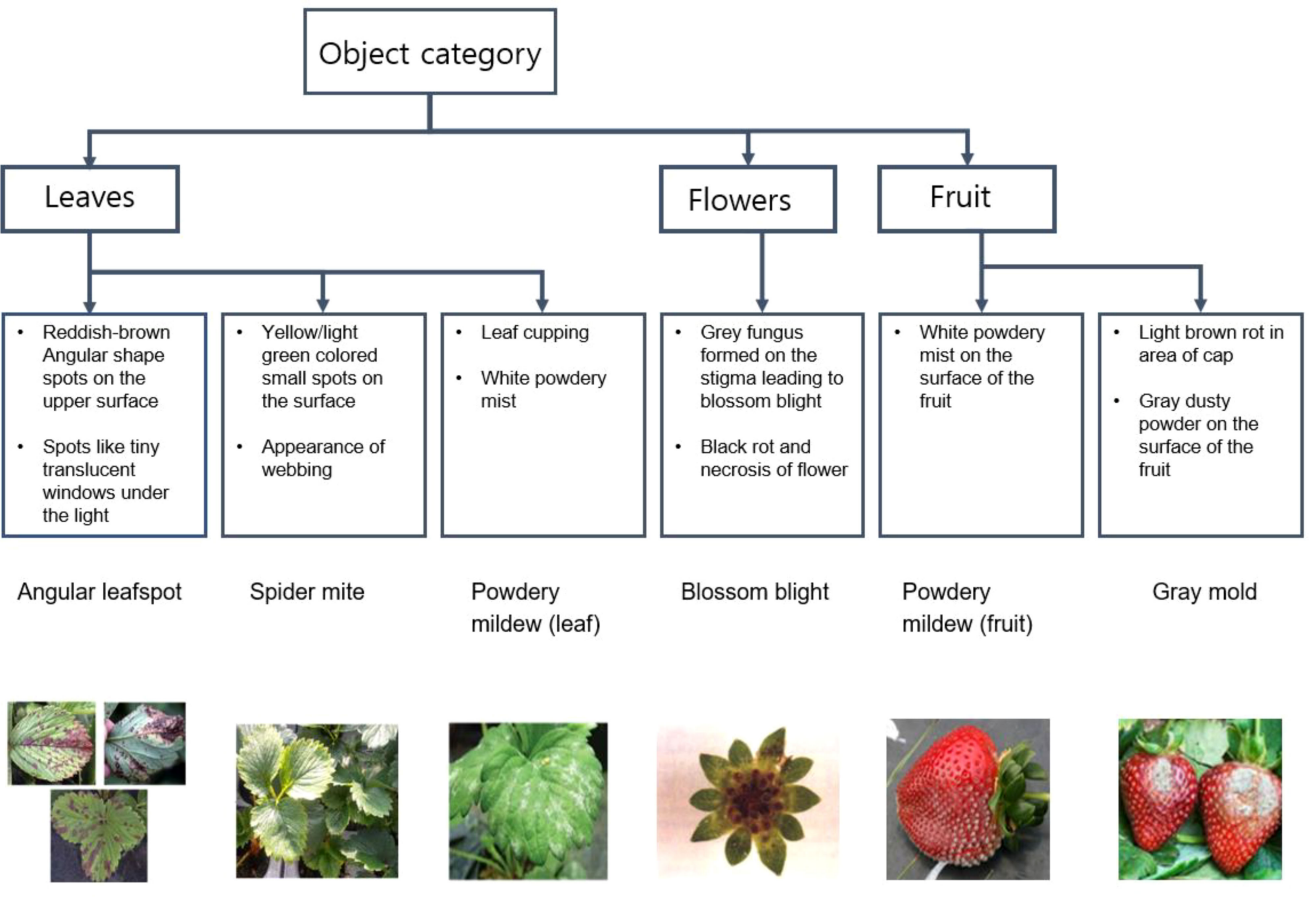
Diagnostic strategy from visual symptoms for angular leaf spot, spider mite, powdery mildew, blossom blight and gray mold rot identification in strawberry plants.

**Figure 5:**
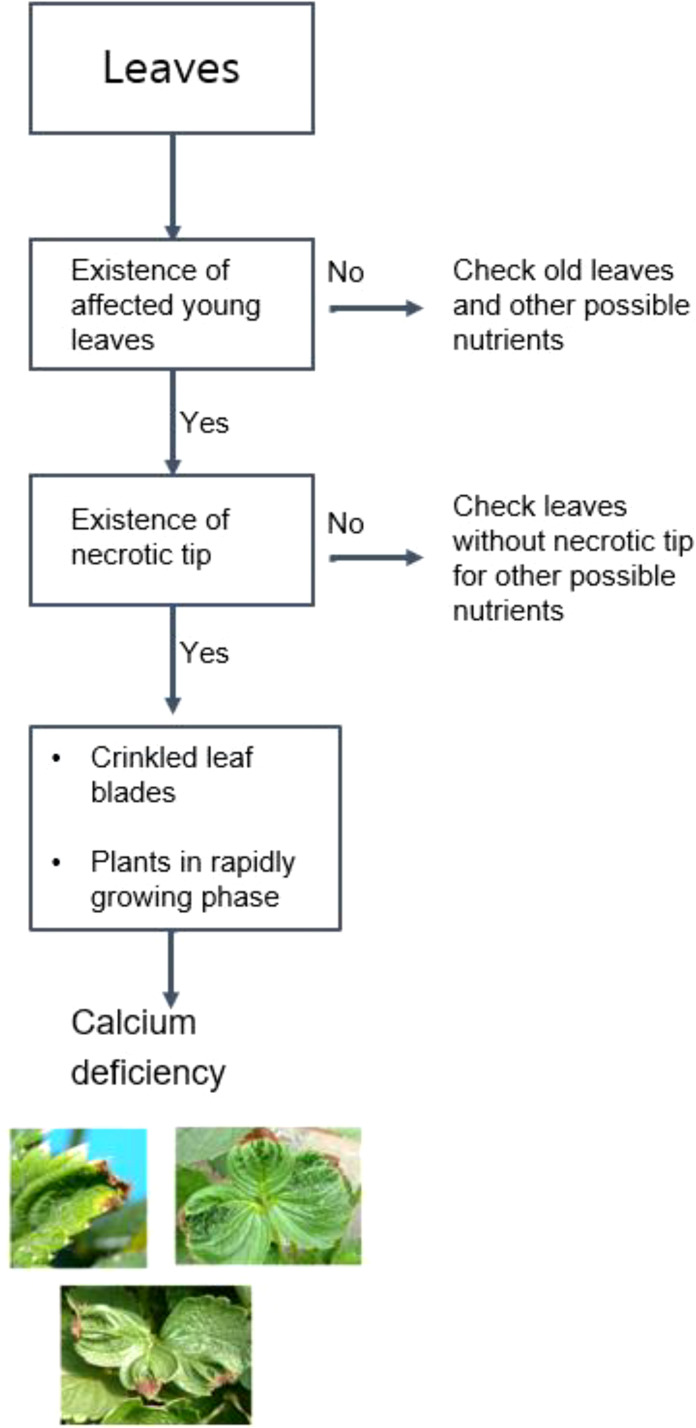
Diagnostic flow-chart for nutrient stress identification in strawberries.

For preliminary identification, a scheme for diagnosing the pest/disease along with stress symptoms observed due to nutrient deficiency in plants was prepared. **Figures 4**, **5** provide the details of the preliminary criteria for diagnosing pest/disease and nutrient stress respectively.

It is difficult to find early symptoms because the visual features to determine a disease or pest infestation level are subtle in the early stages. This subtlety makes the early detection task challenging, so we tried to overcome it by training both the control dataset and the disease/pest dataset. We annotated not only objects of strawberry plants with disease or pests but also leaves, flowers, and fruit of healthy strawberry plants. However, it is hard to annotate every object shown in an image because strawberry plants have short stems, dense trifoliate leaves, and are close to the ground. These features of strawberry plants make some objects occluded by other objects. We excluded objects that occluded over 50% from the annotation to enhance the performance of the training and to filter out the possible regions of outliers. These excluded objects might be shown in other images after the camera slightly moved on. This manual annotation process is laborious and takes a lot of time, so we carried out the process parallel with automatic data acquisition. From Jan 2020 to July 2020, we accumulated a total of 13,393 images of various health conditions and environments. **Table 2** shows some statistics about the data.

**Table 2:** Data statistics.

Label	Number of objects	Number of objects (test data)
Normal	98,904	8,090
Spider mite	12,081	1,217
Calcium deficiency	1,805	188
Angular leafspot	761	58
Blossom blight	714	83
Gray mold	1,373	143
Powdery mildew (fruit)	568	59
Powdery mildew (leaf)	4,260	429

### Data augmentation

We carried out a data augmentation process to prevent overfitting while training and to increase the robustness of the model to make it applicable to images taken in various situations out of our data acquisition conditions. As we have a large number of plant images, it is a burden to save every hard copy of augmented images. Therefore, we used online data augmentation method using image transform functions provided by the PyTorch package (Paszke et al., 2017). We applied random crop, grayscale, horizontal flips, vertical flips, and rotations for the augmentation. We generated modified images by cropping an image from the original one, which width and height ranging from 0.5 to 0.8. After the cropping, we randomly adjusted hue and saturation with displacement ranging ±0.5, and ±0.2 respectively. Varying hue and saturation values reflect the color of the artificial lighting system of strawberry farms. Horizontal and vertical flips are randomly applied. And then images are randomly rotated up to 45-degree.

### Detection model

There are various machine learning models for various image analysis tasks. For the first step, we selected the most suitable image analysis model. It is important to find an adequate part of the strawberry plant to detect early signals of plant diseases and pests. For example, we should focus on the leaves to detect calcium deficiency because the early symptoms of calcium deficiency are tip burn in leaves and cupped and distorted leaves. We took advantage of the deep learning model called ‘You Only Look Once’ (YOLO). This model is one of the most widely-used object detection models because it achieves good detection performance with high speed with relatively light computing power (Redmon et al., 2016). Due to these advantages, many studies on plant diseases and pests detection employed this model (Liu and Wang, 2020; Morbekar et al., 2020; Midhun et al., 2021). The detection module should be installed on the data acquisition system for real application, so considering  omputing power of Jetson TX2 installed on the system, the model should be light enough to run with its environment and should be fast enough to investigate plant images from the camera in real-time manner.

**Figure 6:**
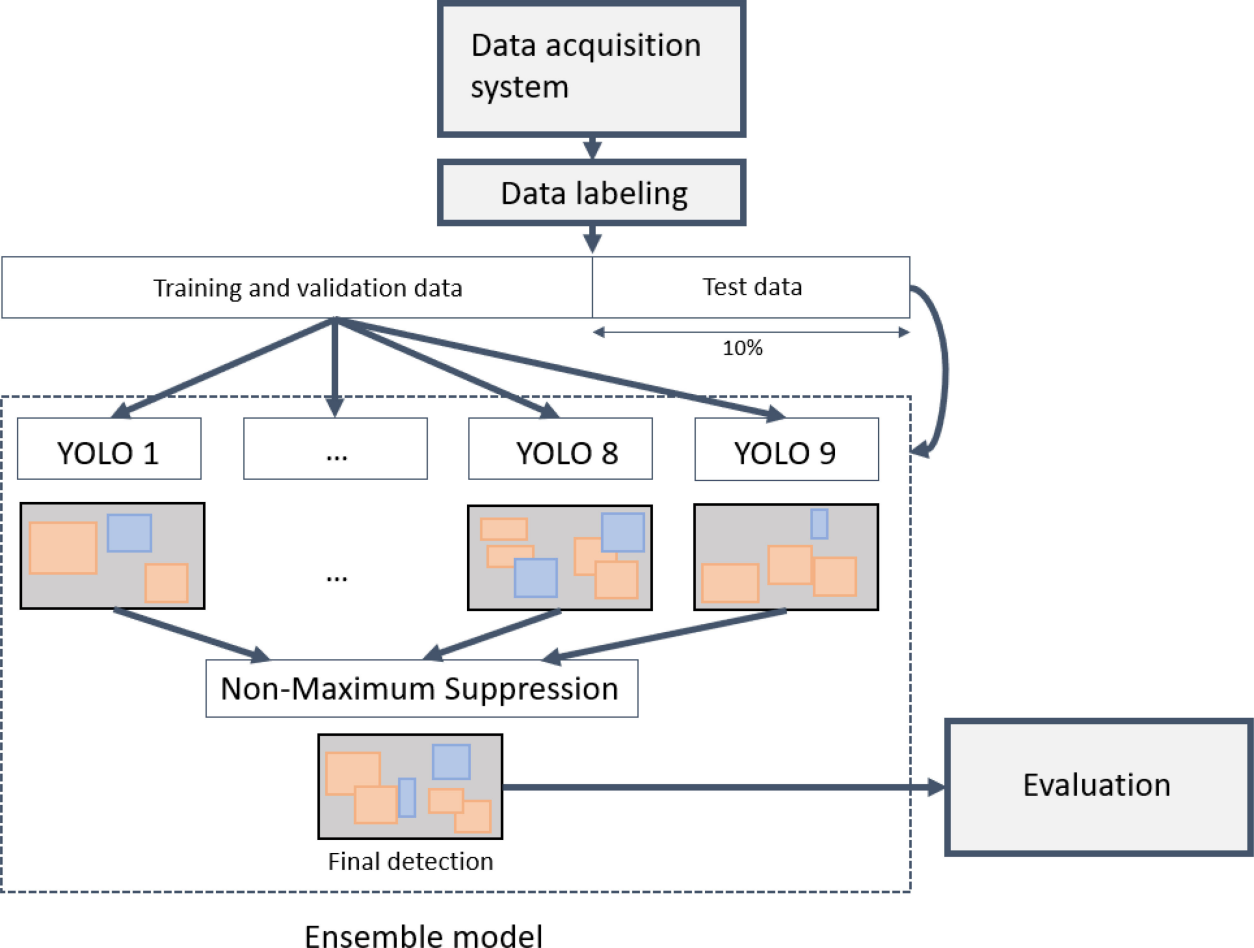
Overall process from data acquisition to model training and evaluation.

The YOLO model calculates a bounding box, predicted label, and a confidence score for every object in an input image. The bounding box is a set of coordinates of the upper-left point and the lower-right point of the object showing information on the location of the object. The predicted label is the result of the prediction of the object, in this case, leaves, flowers, fruits, leaves with early calcium deficiency symptoms, and so on. The prediction is based on the confidence score calculated inside the YOLO model, which is represented as a number between 0 and 1. Like many deep-learning-based models, the changes in hyper-parameters can largely affect the performance of object detection and predictions. So, we applied an ensemble methodology by generating several YOLO models to overcome the local minima problem of machine learning and minimize the effect of hyperparameters from one model. (**Figure 6**; **Supplementary Table 1**) An ensemble model is a widely-used approach to enhance the performance and the robustness of the model (Korez et al., 2020; Xu et al., 2020)

We trained nine YOLO models varying their network size and training datasets using Python version 3.9.7 and PyTorch package version 1.10. For model training, we built training PC with GPU device NVIDIA Tesla V100 with driver and cuda version 11.1. All images were used for training and validation. In every training, we randomly selected 90% of the data as a training and a validation set and the rest of 10% as a test set. For each network size and training dataset, hyperparameters such as learning rate, and weight decay were heuristically determined, and batch size and epoch was set to 64 and 300 respectively (**Supplementary Figure 2**). We used the early stopping algorithm with patience of 50 to reduce the training time, which means the training stops if there is no improvement in last 50 epochs. We got multiple bounding boxes, predictions, and confidence scores for each object as a result of the ensemble model. Non-maximum suppression algorithm was used to combine these multiple results (Neubeck and Van Gool, 2006). It is generally applied to the last part of an object detection model but also can be used to combine results from many models. NMS is a technique to find the best bounding box for each object shown in the image by iteratively calculating intersections of bounding boxes and comparing their labels and confidence scores. After combining overlapping bounding boxes into one bounding box, the predicted label of the bounding box was determined. We determined the predicted label which the sum of confidence scores is the highest.

## Results and discussion

We evaluated the ensemble model with plant images that are not used in the training stage. For object detection evaluation metrics, AUPRC is known to be more suitable than any other metric for an unbalanced dataset that contains a large number of true negatives such as biomedical data. Because our dataset contains a large number of normal objects compared to positive cases, we chose AUPRC as an evaluation metric (Brice and Fabien, 2015; Arun et al., 2020).

**Figure 7:**
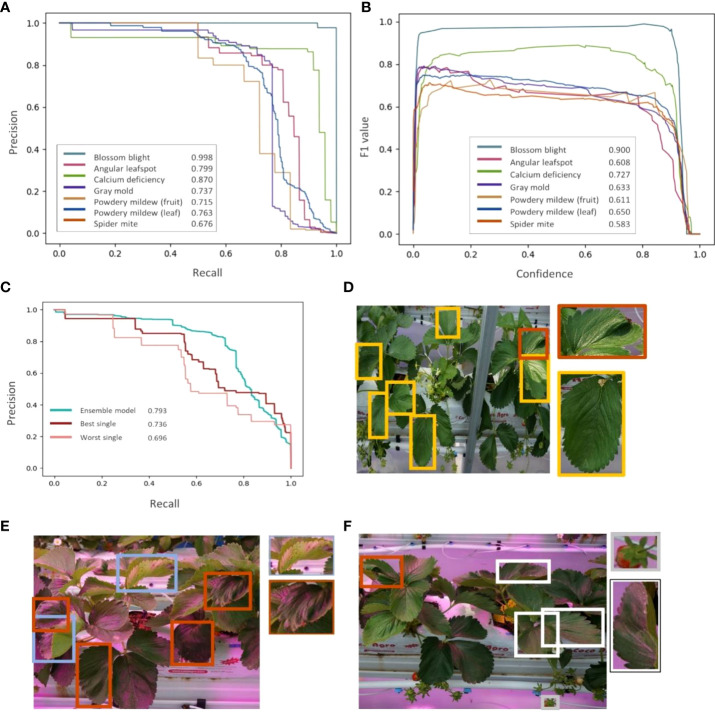
Model training results. **(A)** Precision-Recall curve of seven strawberry diseases and pests. **(B)** F1-curve of seven strawberry diseases and pests. **(C)** Comparison of ensemble model and two single models (best and worst). **(D–F)** An example of detection result of the ensemble model. Orange boxes represent the calcium deficiency cases, **(D)** yellow boxes represent the spider mite cases, **(E)** skyblue boxes represent angular leafspots, **(F)** white boxes represent powdery mildew cases, and a gray box represent the gray mold predicted by the model.

The Area Under Precision-Recall Curve (AUPRC) and the F1 score are used for the evaluation. (**Figure 7**) The model shows performance over 0.67 for all classes and the average AUPRC of 0.793, (**Figure 7A**) which indicates that the model can detect labeled diseases and pests with a decent level. The baseline of AUPRC is the ratio of positive cases to the total objects, so it depends on the test dataset. In this study, leaves, fruits and flowers can be considered as objects, and positive cases are objects in diseases or pests. For example, the baseline for spider mite detection is about 0.12, which is a division of the number of spider mite leaves by the number of normal leaves. (**Table 2**) F1 score is a harmonic mean of precision and recall. F1 curve (**Figure 7B**) is drawn by calculating F1 scores corresponding to the confidence level. We used the integrated F1 score (Powers David, 2011) to evaluate the object detection model. Similar to the result of AUPRC metric, the F1 score for detecting blossom blight is the highest and that for detecting spider mite is the lowest. Comparing every single detection model, an ensemble model shows improved performance in AUPRC (**Figure 7C**). Detection performance for objects with low confidence score is higher in single detection models than in the ensemble model, it may indicate that results from the individual models apt to discord are hard to be integrated for low confident objects.

In both AUPRC and F1 score metrics, the blossom blight shows the highest detection performance. The features of strawberry fruits such as color and shape are clear and distinct compared to the features of leaves. It makes the model sensitive to the small changes in fruit caused by the blossom blight and makes the model detect these changes with high performance. On the other hand, powdery mildew of fruit and gray mold shows lower detection performance than the blossom blight. The early signs of these diseases are similar, showing the small gray spores on the surface of fruits. In the case of leaves, the performance of spider mite detection is lower than that of calcium deficiency. This may be caused by the features of the major symptom that change by the disease or pests. For example, yellow dots on the leaf are the early sign of spider mite (**Figure 7D**) and it can be interrupted by environmental factors such as external lights and the model can mischaracterize yellow dots that are not caused by the pest, for example, the pollen. However, one of the major early signs of calcium deficiency is tip burn and leaf distortion, which are shape-related features and less affected by external lights.

## Conclusions

Early identification of strawberry pests and diseases along with any abnormalities due to nutrient deficiencies are critical for a farmer to avoid huge economic losses and maximize farm productivity along with ensuring good crop quality. Developing quick, accurate and easy to use practical plant disease and stress detection technique was a major objective of this current study. One of the challenging points of the early detection of plant diseases is the lack of relevant image data. As plant diseases and pests progress fast since their first infection, it is hard to collect their early images manually. Also, to install the trained detection module directly to the device for further applications, it is needed to standardize the features of the image such as distances from the subjects.

We built an automatic system for plant data acquisition to overcome the above. In this study, a camera module was applied to the system to collect a large amount of plant image data, and collected about 13,000 images with nearly 120,000 objects. The system can also be used to collect other kinds of data, for example, by installing ultrasonic sensors or infrared sensors, we can focus on various aspects of plant status, or with spray modules, this system can be further applied to controlled experiments.

We trained an object detection model based on the YOLO v5 structure with accumulated data from the acquisition system. However, the performance was not satisfactory because the features of early infections are subtle. So to improve the detection performances, we built an ensemble detection model using nine independent models. The model detects early signs of common strawberry diseases and pests using images taken from the bed with decent performances represented by AUPRC, 0.819 of its average.

Aforementioned imaging system and deep learning-based detection module can monitor a large number of strawberry plants periodically and automatically and can notify users if there are any signs of early symptoms of diseases or pests by connecting with user-side devices. Also, this data acquisition and detection model training workflow can be applied to not only diseases and pests covered in this study but also other important strawberry diseases, pests and conditions such as iron deficiency and phosphate deficiency, with continuous data accumulation and labeling.

## Data availability statement

The raw data supporting the conclusions of this article will be made available by the authors, without undue reservation.

## Author contributions

SL performed data collection and analysis, AA managed the plant experiment environment, and CY designed the hardware system. All authors contributed to building a data acquisition system, reviewed the results, and prepared the manuscript. All authors contributed to the article and approved the submitted version.

## Funding

This work was supported by Korea Institute of Planning and Evaluation for Technology in Food, Agriculture and Forestry (IPET) and Korea Smart Farm R&D Foundation (KosFarm) through Smart Farm Innovation Technology Development Program, funded by Ministry of Agriculture, Food and Rural Affairs (MAFRA) and Ministry of Science and ICT (MSIT), Rural Development Administration (RDA)(421008-04).

## Conflict of interest

Author CY is the CEO of the company Sherpa Space. Inc., and author AA is employed by Sherpa Space. Inc.

The remaining authors declare that the research was conducted in the absence of any commercial or financial relationships that could be constructed as a potential conflict of interest.

## Publisher’s note

All claims expressed in this article are solely those of the authors and do not necessarily represent those of their affiliated organizations, or those of the publisher, the editors and the reviewers. Any product that may be evaluated in this article, or claim that may be made by its manufacturer, is not guaranteed or endorsed by the publisher.
