# Supplementary material for: Detecting strawberry diseases and pest infections in the very early stage with an ensemble deep-learning model
Source: Front Plant Sci. 2022 Oct 12;13:991134. doi: 10.3389/fpls.2022.991134 (PMC9597313; doi:10.3389/fpls.2022.991134)
Supplement: Supplementary file 1 [file DataSheet_1.pdf]

Supplementary material: Detecting strawberry diseases and pest infections in the very early stage with an ensemble deep-learning model

Sangyeon Lee, Amarpreet Singh Arora, and Choa Mun Yun[*]

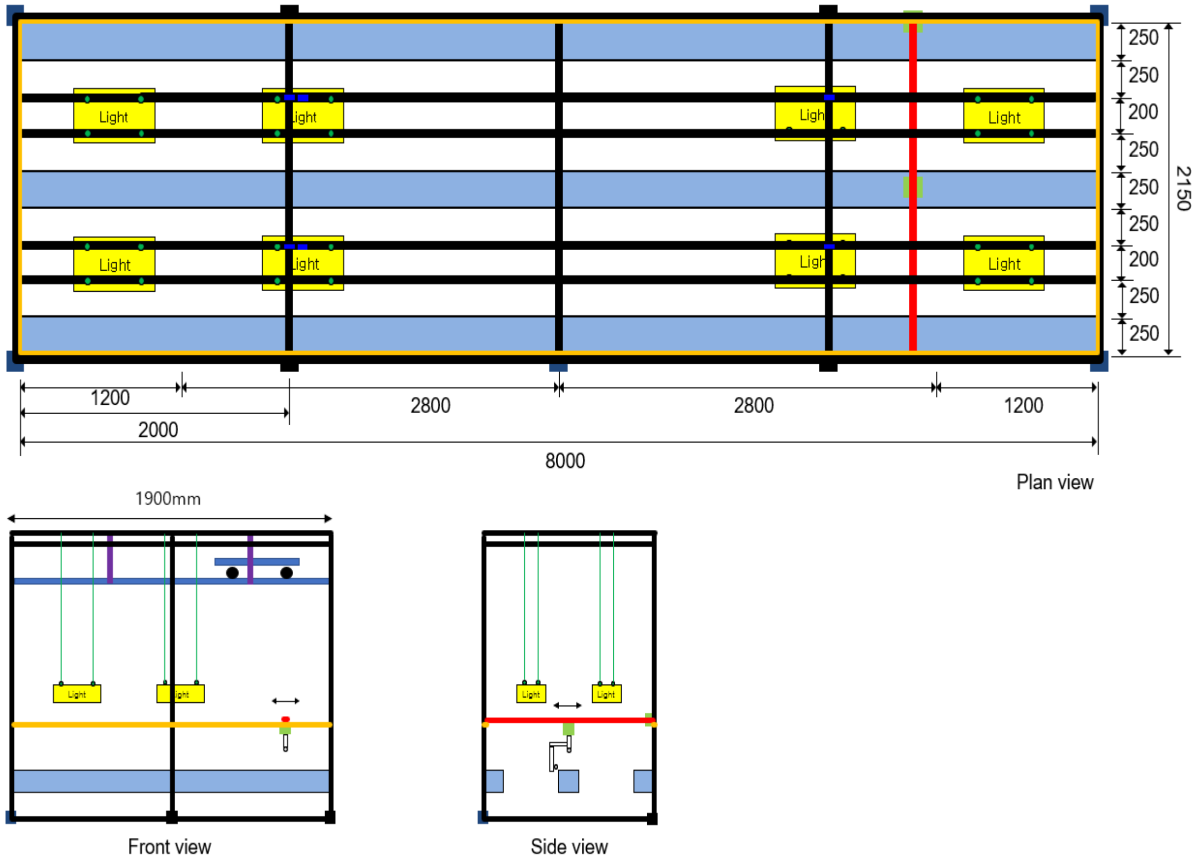

Supplementary figure 1. Detailed plan of hardware of data acquisition system, plan view, front view and side view. Every numbers representing width, length and height are millimeter (mm) scale. Main frames are represented as black lines, strawberry beds as blue boxes, motion stage and rail as yellow lines, a bar for a moving belt and a camera as a red line. Yellow boxes are artificial lighting devices.

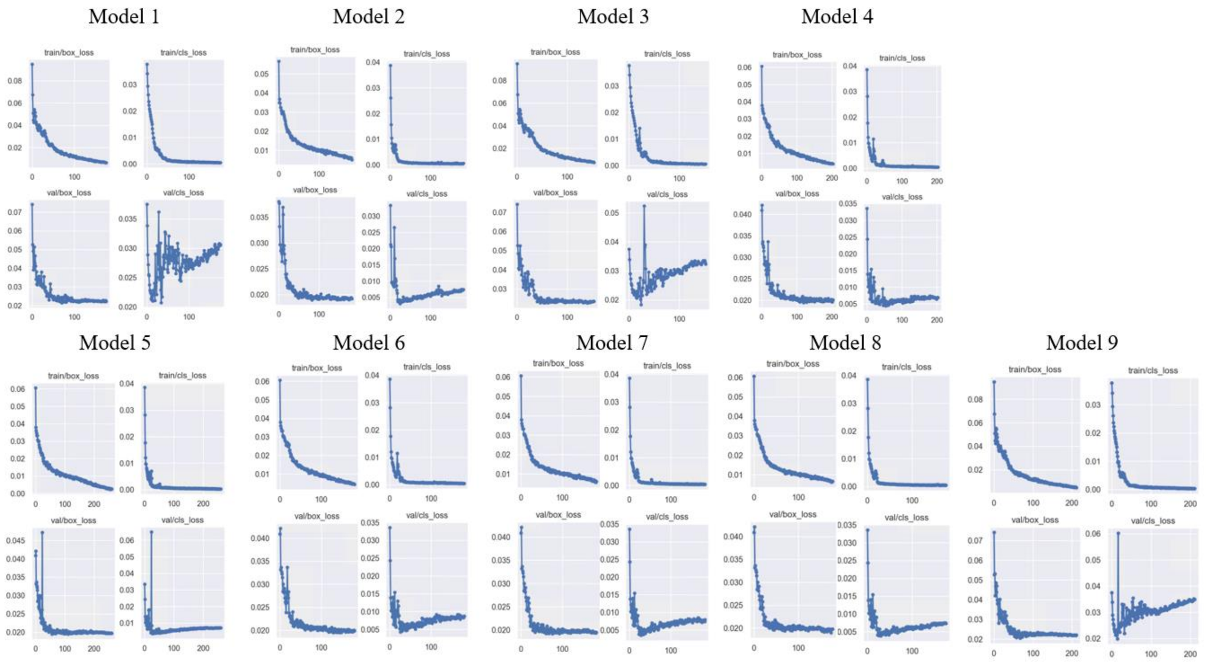

Supplementary figure 2. Decrease of losses of every single object detection model. Four losses for each model: upper left: training box loss, upper right: training class loss, lower left: test box loss, lower right: test class loss. The box losses decrease continuously and converge, indicating that every model is capable to detect labeled objects (leaves, fruits and flowers). However, class losses in some models tend to converge to the higher value than its minimum value, means some classes of diseases/pest infections shows similar visual features in its initial stage.

Supplementary table 1. Hyper-parameters of individual object detection models used for an ensemble model.

We choosed the input image size (width and height) between 640 and 960. Bigger than input size 960 pixel raises the calculation burden and smaller than 640 decreases the resolution and performance. Learning rate and weight decay are determined as a result of grid search. Hyper-parameter candidates of learning rate are: 5e-2, 1e-2, 5e-3, 1e-3, 5e-4, 1e-4, 5e-5, 1e-5, 5e-6 and 1e-6 and candidates of weight decay are: 1e-2, 1e-3, 1e-4, 1e-5, and 1e-6. Hyper-parameters are selected corresponding to their validation loss, top 3 were taken for each YOLO structure. [1]

MODEL	Structure	Input size	Learning rate	Weight decay
YOLO 1	yolo v5 s	640	1e-5	1e-3
YOLO 2	yolo v5 s	640	5e-4	1e-4
YOLO 3	yolo v5 s	960	1e-4	1e-3
YOLO 4	yolo v5 m	640	1e-5	1e-3
YOLO 5	yolo v5 m	960	5e-4	1e-4
YOLO 6	yolo v5 m	960	5e-4	1e-5
YOLO 7	yolo v5 l	960	1e-5	1e-5
YOLO 8	yolo v5 l	960	5e-4	1e-5
YOLO 9	yolo v5 l	960	1e-4	1e-4

Reference

1. Glenn Jocher, Ayush Chaurasia, Alex Stoken, Jirka Borovec, NanoCode012, Yonghye Kwon, TaoXie, Jiacong Fang, imyhxy, Kalen Michael, Lorna, Abhiram V, Diego Montes, Jebastin Nadar, Laughing, tkianai, yxNONG, Piotr Skalski, Zhiqiang Wang, … Mai Thanh Minh. (2022). ultralytics/yolov5: v6.1 - TensorRT, TensorFlow Edge TPU and OpenVINO Export and Inference (v6.1). Zenodo. https://doi.org/10.5281/zenodo.6222936
